# Retraction: Is Refracture a Concern Following Closed Management of Tibia Shaft Fractures in Children? 

**DOI:** 10.7759/cureus.r18

**Published:** 2020-03-19

**Authors:** Eli Ahdoot, Ryne Jenkins, Theresa Pak, Henry Tsang, Juston Fan

**Affiliations:** 1 Orthopaedic Surgery, Riverside University Health System Medical Center, Moreno Valley, USA; 2 Orthopaedic Surgery, Riverside University Health System Medical Center, Moreno Valley , USA

This article has been retracted after a request made by the authors due to an unresolvable authorship dispute. After conducting our own investigation, Cureus has made the decision to formally retract this article.

